# Individual and Joint Impacts of Ethanol Use, BMI, Age and Gender on Serum Gamma-Glutamyltransferase Levels in Healthy Volunteers

**DOI:** 10.3390/ijms140611929

**Published:** 2013-06-04

**Authors:** Joanna Danielsson, Päivikki Kangastupa, Tiina Laatikainen, Mauri Aalto, Onni Niemelä

**Affiliations:** 1 Department of Laboratory Medicine and Medical Research Unit, Seinäjoki Central Hospital, and University of Tampere, Seinäjoki 60220, Finland; E-Mails: joanna.danielsson@epshp.fi (J.D.); paivikki.kangastupa@epshp.fi (P.K.); 2 National Institute for Health and Welfare (THL), Helsinki 00271, Finland; E-Mails: tiina.laatikainen@thl.fi (T.L.); mauri.aalto@epshp.fi (M.A.); 3 The Institute of Public Health and Clinical Nutrition, University of Eastern Finland, Kuopio 70211, Finland; 4 Hospital District of North Karelia, Joensuu 80210, Finland; 5 Department of Psychiatry, Seinäjoki Central Hospital, Seinäjoki 60220, Finland

**Keywords:** alcohol, oxidative stress, ageing, obesity, liver

## Abstract

Excessive ethanol consumption, obesity and increasing age may all lead to increased serum levels of gamma-glutamyltransferase (GGT) enzyme, which plays a key role in the metabolism of extracellular reduced glutathione. However, as yet, the interactions between the various modulators of GGT activities have remained poorly defined. We analyzed data from 15,617 apparently healthy individuals (7254 men and 8363 women, mean age 46 ± 13 years, range 25–74 years) who participated in a national cross-sectional health survey in Finland between 1997 and 2007. All subjects underwent detailed clinical examinations and interviews, including the amount of ethanol use and smoking habits. GGT levels were measured from all participants, and the individual and joint impacts of the different study variables on GGT levels were assessed. Significant individual effects were noted for ethanol use (*p* < 0.001), body mass index (BMI) (*p* < 0.001), age (*p* < 0.001) and smoking (*p* < 0.001). In men, significant two-factor interactions occurred between ethanol use and age (*p* < 0.020). Among those over 40 years of age, ethanol consumption was found to be a stronger determinant of increased GGT levels than in men below 40 years, whereas in the latter age group, BMI was found to predominate. In women, a significant two-factor interaction occurred between ethanol and BMI (*p =* 0.010), whereas it did not with ethanol use and age. The data underscores the role of ethanol consumption and age as major determinants of increased GGT levels in men, whereas in women, a relatively stronger impact was noted for ethanol intake and BMI. In light of the ability of GGT enzyme to modulate crucial redox-sensitive functions, the present findings also support the use of GGT as a biomarker of oxidative stress.

## 1. Introduction

The increasing prevalence of excess ethanol intake constitutes a major threat to modern healthcare [1–3]. During the past decades, obesity and its comorbidities have also become a worldwide epidemic, more than half of the population being currently overweight or obese [4–7]. Both excessive ethanol consumption and obesity are also well known causes of hepatic fat accumulation and increased blood levels of gamma-glutamyltransferase (GGT) enzyme [8,9], which is a key modulator of glutathione (GSH) metabolism and oxidative stress [10,11].

Consequently, there has been growing interest in the biological significance of alterations in serum GGT, which has been suggested to be useful as a general indicator of oxidative stress [10,12]. GGT levels may also provide prognostic health information in a wide variety of diseases of the modern life [13]. The expression of GGT is increased as an adaptive response upon exposure to oxidative stress, and mild elevations of GGT apparently relate to its biological function to maintain intracellular levels of GSH [11]. In individuals with high ethanol intake and excess body weight, slight to moderate increases in GGT levels appear to mark enhanced oxidative stress burden [10,14]. A trend towards increased mean GGT levels has been observed in recent population surveys in connection with excess ethanol consumption and increased prevalence of obesity [14–16]. However, so far, studies on the interactions between ethanol intake, overweight, age and gender on GGT levels have been limited.

In this work, we investigated the individual and joint impacts of ethanol use and body mass index (BMI) on GGT levels in a large age- and gender-stratified population sample of teetotalers, moderate drinkers and individuals exceeding the limits of heavy drinking, yet being devoid of apparent health problems at the time of the study.

## 2. Results

**Figure 1:**
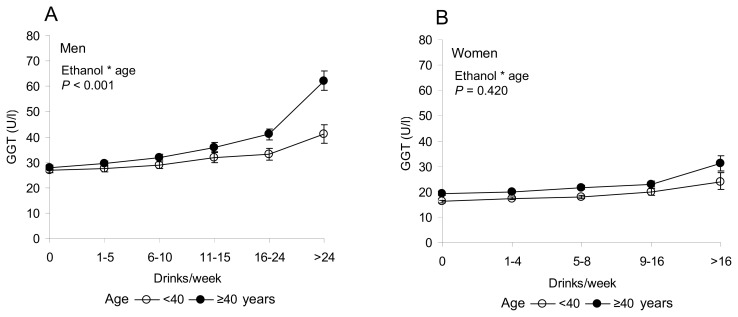
Gamma-glutamyltransferase (GGT) levels (mean ± 95% confidence interval (CI)) in men (**A**) and women (**B**) in subgroups divided according to the amount of ethanol intake and age. (**A**) In men, there was a significant two-factor interaction between ethanol use and age (*p* < 0.001). In men over 40 years of age, GGT levels increased sharply after ethanol consumption exceeded 16 drinks per week. (**B**) In women, the interaction between ethanol and age was not significant. BMI (kg/m^2^) and smoking (cigarettes/day) were used as covariates.

Main characteristics of the 15,617 study subjects classified according age, gender, ethanol consumption, BMI and smoking are presented in Table 1. In the total population, there was a significant individual effect of ethanol use (*p* < 0.001), body mass index (*p* < 0.001), age (*p* < 0.001) and smoking (*p* < 0.001) on serum GGT levels. In men, a significant two-factor interaction was noted between ethanol use and age (*p* < 0.020). Among men over 40 years of age, ethanol consumption was found to be a markedly stronger determinant of increased GGT levels than in men below 40 (Figure 1A). In women, the interaction between ethanol intake and age was not found to be significant (Figure 1B).

**Figure 2:**
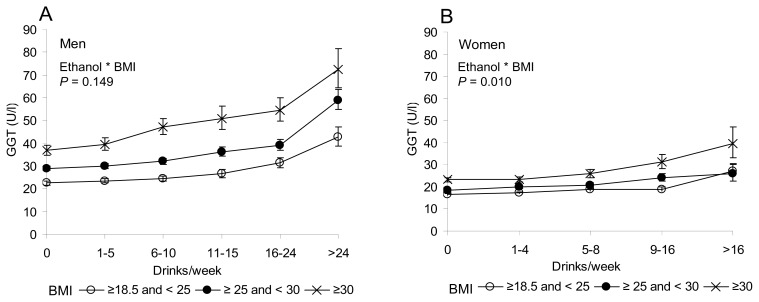
GGT levels (mean ± 95% CI) in men (**A**) and women (**B**) as divided to subgroups according to the amount of ethanol intake and BMI. (**A**) In men, the interaction between ethanol and BMI was not significant (*p =* 0.149). (**B**) A significant two-factor interaction (*p =* 0.010) between ethanol and BMI was noted among women. Age (years) and smoking (cigarettes/day) were used as covariates.

Two-factor analyses on ethanol and BMI indicated a significant interaction among women (*p =* 0.010), whereas not in men (*p =* 0.149) (Figure 2). Obese women (BMIs above 30) were found to be more prone towards increased GGT levels upon increasing ethanol intake than the other BMI-based subgroups of women.

In the present material, multiple linear regression analyses showed multivariable adjusted beta coefficients of 0.22 for GGT *versus* ethanol intake and 0.26 for GGT *versus* BMI (adjusted for age, sex and smoking). Table 2 summarizes the correlations between GGT, ethanol intake and BMI in different age groups. In men, the correlations between ethanol consumption and GGT were found to be significantly different between the younger and older age groups, the latter showing significantly stronger correlations. In women, the correlations between GGT and ethanol intake in the different age groups were not different. The correlations between GGT and BMI were also found to vary according to age, the younger subjects showing significantly stronger associations (Table 2).

## 3. Discussion

Our study in a large and nationally representative sample of apparently healthy individuals with various levels of ethanol consumption indicates distinct age- and gender-dependent interactions between ethanol intake, BMI and serum GGT, which has recently been proposed as a biomarker of oxidative stress [10,12]. In men, especially in those over 40 years of age, ethanol consumption appeared to be the major determinant on GGT levels, whereas in women, increased BMI was found to influence GGT activities in a more predominant manner.

Both excess ethanol consumption and obesity seriously threaten health and wellbeing in our societies. In Finland, ethanol consumption currently exceeds 10 L of absolute ethanol per capita, and almost 70% of working aged men and 50% of women are overweight, 30% of both genders being obese [17]. Consequently, ethanol and obesity-related health problems are frequently co-existing phenomena, which can also create supra-additive effects on serum liver-derived enzyme activities [9,14,18]. In obese subjects, elevated serum liver enzymes have been observed even as a result of ingesting small to moderate amounts of ethanol on a regular basis [9,14]. Among men, ethanol consumption has also been shown to interact with smoking in increasing GGT [19]. However, studies on the interactions between various health risk behaviors and serum GGT have, so far, been limited.

In accordance with our recent observations [20], notable changes in GGT levels were seen after ethanol consumption exceeded four (women) or eight (men) standard drinks per week. Mild elevations of GGT in such individuals could be considered a sign of a need to maintain the intracellular levels of glutathione [21]. In men over 40 years of age, GGT appeared to increase at ethanol consumption levels, which were only about half of those found in the age group below 40, suggesting that aging may render individuals more susceptible to oxidative stress and ethanol-induced adverse health consequences [20,22]. In agreement with this view, Loomba *et al.* [23] have previously reported that a combination of obesity and ethanol intake increases the risk of liver injury, especially in older individuals. Obviously, safe limits for ethanol intake in individuals with advanced age and in those with increased body weight need further attention. Interestingly, previous studies have further indicated that the association between GGT levels and all-cause mortality is also age-dependent, which may in part be due to age-related differences in the ability of maintaining homeostasis and clearing xenobiotics [12,24]. There may also be differences with respect to health risks of being overweight, whether developed in early adulthood or later in life [25].

Currently, the pathophysiological mechanisms linking adverse health effects of ethanol consumption, obesity and increasing age are poorly known. Even single bouts of heavy drinking may lead to hepatic fat accumulation and a rise in blood GGT enzyme levels [26]. Ethanol has a high energy content, and in experimental animals, ethanol-induced oxidative stress has been shown to be aggravated by high-fat-diets [27]. Ethanol administration to rats leads to enhanced GGT activities and a loss of GSH into the circulation [28,29]. GGT activation also seems to be related with the development of superoxide ion, hydrogen peroxide, unintended oxidation of lipoproteins and generation of a proinflammatory status in the body [30].

Due to the fact that GGT plays a pivotal role in GSH metabolism and the antioxidant defense system, several lines of both experimental and clinical evidence have proposed a role for GGT as a biomarker for oxidative stress [10,12–15,28,29,31–33]. While assays of more specific indicators of oxidative stress were beyond the scope of the present study, it should be noted that in previous studies, GGT has shown a correlation with 8-hydroxydeoxyguanosine (8-OHdG), acrolein-lysine adducts and aldehydic products of lipid peroxidation [34–37]. In humans, increased GGT levels have been reported in a wide variety of chronic diseases, and several lines of investigation have further emphasized the role of GGT as a risk marker for conditions involving oxidative stress, including cancer, neurodegenerative diseases, rheumatoid arthritis, atherosclerosis related events, fatty liver disease, diabetes and metabolic syndrome [12,13,38–46]. While among women, high GGT levels have been suggested to predict carcinogenesis [40], men seem to show increased cardiovascular mortality risks from elevated GGT levels, especially when there is simultaneous evidence of hepatic steatosis [47]. In the presence of one etiology (ethanol or obesity) strong enough to induce fatty liver, generation of additional oxidative stress brought about by a possible second etiology is also expected to increase the risk for progressive liver damage [21,48,49]. It remains to be established in future studies whether (i) this theory of two hits could also be generalized to health risks outside the liver and (ii) whether GGT could also be a mediator between the hepatic and extrahepatic health risks [50].

The present findings should be implicated in the clinical use of GGT as a biomarker of ethanol intake, oxidative stress and liver dysfunction. Although, it has been widely accepted that through more effective early diagnosis, the prevalence of diseases due to high risk health behaviors can be significantly reduced [2,3], lack of knowledge on the relationships between ethanol, obesity and GGT levels and inconsistencies regarding the definition of safe levels of ethanol intake in different populations have complicated such efforts. The present data provides novel information on the factors influencing early-stage GGT activation and shows that the doses of ethanol required to reach significant GGT elevation varies markedly as a function of age, BMI and gender. Our findings also emphasize the need for recalibrating goals for GGT normal ranges in populations with different demographic characteristics. Similarly, current data should also be considered in the definitions of safe limits of ethanol intake in such populations.

## 4. Subjects and Methods

### 4.1. Study Protocol

Three independent, cross-sectional population health surveys (The National FINRISK Studies) were carried out in six geographic areas in Finland in the years 1997, 2002 and 2007. In each year, an age- and a gender-stratified random sample of persons 25 to 74 years of age was drawn from the population register. The survey included a detailed questionnaire on health status and ethanol intake, physical measurements and laboratory tests. The self-administered questionnaire covered information on current health behavior, health status, medical history and socioeconomic factors. The medical examinations were conducted using a standardized protocol of the WHO MONICA project [51] and the more recent recommendations of the European Health Risk Monitoring project [52]. Measurements of height and weight were carried out, and body mass index (BMI, kg/m^2^) was calculated as a measure of relative body weight. All surveys were conducted in accordance with the Declaration of Helsinki according to the ethical rules of the National Public Health Institute. The approvals for the FINRISK surveys were applied each time from relevant ethics committees.

Serum GGT (U/L) was measured by standard kinetic methods following recommendations of the European Committee for Clinical Laboratory Standards (ECCLS) [53] using an Abbott Architect clinical chemistry analyzer (Abbott Laboratories, Abbott Park, IL, USA) showing the following analytical characteristics: intra- and inter-assay coefficients of variation of 1.2% and 2.3%, respectively; mean measurement bias of 4.2%; measurement range of 4–1540 U/L, the lower limit of detection being 4.0 U/L.

The present sample included data from the subjects who both filled out the questionnaire and attended the medical examination. The response rates ranged from 64% to 71%. In order to obtain a representative sample of apparently healthy individuals, exclusions were made for the following reasons: pregnancy (*n =* 137), diagnosis of myocardial infarction (*n =* 627), stroke, cerebral hemorrhage or embolism (*n =* 517), diabetes or glucose-intolerance (*n =* 1137), hypertension (*n =* 2765), use of statins or lipid lowering agents (*n =* 1037) or signs of active infection at the time of the study (*n =* 837). In addition, exclusions were made due to missing variables (*n =* 1780). The final population thus comprised 15,617 individuals; 7254 men and 8363 women.

Ethanol consumption was assessed with detailed questions on the type of alcoholic beverage consumed, the frequency of consumption and the amount of ethanol-containing drinks consumed regularly during the past one week and one year prior to sampling. The amount of ethanol in different beverages was quantified as follows: one bottle (330 mL) of beer (12 g), one bottle (330 mL) of strong beer (15.5 g), one bottle (330 mL) of long drink (15.5 g), one standard drink (4 cL) of spirit (12 g), one glass (12 cL) of wine (12 g) and one bottle (330 mL) of cider (12 g). A dose of 12 g of pure ethanol was considered one standard drink. The subjects were subsequently classified into groups as follows: persons who reported no ethanol consumption were referred to as abstainers (*n =* 5250); moderate drinkers (*n =* 9781) consumed less than 280 g of ethanol (men) or less than 190 g of ethanol (women) per week; heavy drinkers (*n =* 586) consumed over 280 g per week (men) or over 190 g per week (women). Smoking was quantified as the amount of cigarettes per day. The population was further divided into subgroups according to body mass index (BMI) and age, as shown in Table 1.

### 4.2. Statistical Methods

Values are expressed as the mean ± SD or the mean ± 95% confidence interval (CI). Comparisons between groups were made with ANOVA using the Bonferroni *post hoc* test for multiple comparisons and appropriate covariates as indicated. Factorial ANOVA was used to investigate the interactions between drinking status, BMI, age and gender on serum GGT. Before the analyses, the data on serum GGT were subjected to logarithmic transformation to yield non-skewed distributions with homogeneity of variance. Correlations were calculated using partial correlation coefficients. The differences between correlations were analyzed with the *Z*-test for correlation coefficients. Multiple linear regression analysis was used to assess the correlates of GGT adjusted for age, sex and smoking. All analyses were carried out with the use of SPSS 19.0 for Windows statistical software (SPSS Inc., Chicago, IL, USA). A *p*-value <0.05 was considered statistically significant.

## 5. Conclusions

Our cross-sectional study indicates that in men over 40 years of age, ethanol consumption is a stronger determinant of increased gamma-glutamyltransferase (GGT) enzyme levels than in men below 40. In women the interaction between ethanol intake and age was not found to be significant, whereas a significant interaction emerged between ethanol and body mass index such that obese women were more prone towards increased GGT levels upon increasing ethanol intake. The data supports distinct age- and gender-dependent impacts of alcohol consumption and excess body weight on serum GGT, which plays a pivotal role in maintaining the intracellular levels of glutathione and could thereby also serve as a biomarker of oxidative stress status in the body.

## Figures and Tables

**Table 1:** Main characteristics of the study population as classified according age, gender, ethanol consumption, BMI and smoking.

	Men, age group					
	
	25–29	30–39	40–49	50–59	60–69	70–74
	
N	799	1747	1854	1586	999	269
Ethanol						

No intake						
*n* (%)	203 (25%)	417 (24%)	464 (25%)	395 (25%)	315 (32%)	111 (41%)

<280 g/week	100 ± 69	102 ± 70	104 ± 70	100 ± 69	82 ± 65	73 ± 60
*n* (%)	556 (70%)	1223 (70%)	1271 (69%)	1072 (68%)	636 (64%)	153 (57%)

>280 g/week	430 ± 175	471 ± 262	482 ± 284	464 ± 201	458 ± 192	483 ± 223
*n* (%)	40 (5%)	107 (6%)	119 (6%)	119 (8%)	48 (5%)	5 (2%)
BMI, kg/m^2^						

Underweight						
<18.5	18.0 ± 0.5	17.8 ± 0.8	17.8 ± 1.0	18.3 ± 0.2	18.4 ± 0.1	17.5
*n* (%)	8 (1%)	8 (0.5%)	5 (0.3%)	3 (0.2%)	2 (0.2%)	1 (0.4%)

Normal weight						
≥18.5 and <25	22.7 ± 1.6	23.0 ± 1.5	23.2 ± 1.4	23.3 ± 1.4	23.1 ± 1.4	23.3 ± 1.4
*n* (%)	437 (55%)	687 (39%)	614 (33%)	493 (31%)	278 (28%)	77 (29%)

Overweight						
≥25 and <30	27.0 ± 1.4	27.0 ± 1.3	27.1 ± 1.4	27.3 ± 1.5	27.2 ± 1.3	27.1 ± 1.3
*n* (%)	282 (35%)	796 (46%)	935 (50%)	784 (49%)	533 (53%)	154 (57%)

Obesity						
≥30	32.8 ± 3.0	33.1 ± 3.0	32.9 ± 2.9	32.7 ± 2.8	32.5 ± 2.4	32.4 ± 2.3
*n* (%)	72 (9%)	256 (15%)	300 (16%)	306 (19%)	186 (19%)	37 (14%)
Tobacco						

No smoking						
*n* (%)	479 (60%)	1132 (65%)	1267 (68%)	1138 (72%)	819 (82%)	233 (87%)

Cigarettes/day	13.4 ± 8.5	15.8 ± 9.0	17.5 ± 9.1	18.2 ± 8.8	17.7 ± 8.7	14.0 ± 7.0
*n* (%)	320 (40%)	615 (35%)	587 (32%)	448 (28%)	180 (18%)	36 (13%)

	**Women, age group**					
	
	**25**–**29**	**30**–**39**	**40**–**49**	**50**–**59**	**60**–**69**	**70**–**74**
	
N	1034	2028	2176	1855	1011	259
Ethanol						

No intake						
*n* (%)	419 (41%)	793 (39%)	786 (36%)	724 (39%)	475 (47%)	148 (57%)

<190 g/week	57 ± 40	53 ± 40	56 ± 42	54 ± 40	47 ± 36	37 ± 31
*n* (%)	607 (59%)	1198 (59%)	1340 (62%)	1090 (59%)	525 (52%)	110 (42%)

>190 g/week	223 ± 20	279 ± 71	293 ± 85	298 ± 131	284 ± 96	300
*n* (%)	8 (0.8%)	37 (2%)	50 (2%)	41 (2%)	11 (1%)	1 (0.4%)
BMI, kg/m^2^						

Underweight						
<18.5	17.8 ± 0.6	17.6 ± 0.7	17.6 ± 0.9	17.9 ± 0.5	17.3 ± 0.9	17.9 ± 0.6
*n* (%)	45 (4%)	33 (2%)	17 (0.8%)	8 (0.4%)	4 (0.4%)	4 (2%)

Normal weight						
≥18.5 and <25	21.8 ± 1.7	22.2 ± 1.6	22.5 ± 1.6	22.8 ± 1.5	22.9 ± 1.5	22.9 ± 1.6
*n* (%)	721 (70%)	1,238 (61%)	1,176 (54%)	752 (41%)	327 (32%)	87 (34%)

Overweight						
≥25 and <30	27.0 ± 1.5	27.1 ± 1.4	27.1 ± 1.4	27.1 ± 1.4	27.3 ± 1.4	27. 3 ± 1.5
*n* (%)	191 (18%)	551 (27%)	674 (31%)	739 (40%)	440 (44%)	103 (40%)

Obesity						
≥30	34.3 ± 3.8	34.2 ± 4.0	33.5 ± 3.0	33.8 ± 3.6	33.2 ± 3.0	33.7 ± 3.1
*n* (%)	77 (7%)	206 (10%)	309 (14%)	356 (19%)	240 (24%)	65 (25%)
Tobacco						

No smoking						
*n* (%)	730 (71%)	1501 (74%)	1644 (76%)	1520 (82%)	900 (89%)	242 (93%)

Cigarettes/day	9.3 ± 6.0	10.8 ± 6.9	11.6 ± 7.1	12.7 ± 7.0	12.9 ± 7.5	11.5 ± 8.5
*n* (%)	304 (29%)	527 (26%)	532 (24%)	335 (18%)	111 (11%)	17 (7%)

Ethanol intake (g/week), BMI (kg/m^2^) and smoking (number of cigarettes/day) are expressed as the mean ± SD. The numbers of observations (*n*) are also given as percentages (%) from the total number of observations in each age group.

**Table 2:** Partial correlations between GGT and ethanol intake or GGT and BMI in different age groups of men and women.

	Men			Women		
		
Age group	GGT *vs.* ethanol intake	GGT *vs.* BMI	*n*	GGT *vs.* ethanol intake	GGT *vs.* BMI	*n*
25–29	0.19	0.45	799	0.14	0.30	1034
30–39	0.20	0.43	1747	0.17	0.25	2028
40–49	0.28 ^*, ††^	0.33 ^***, †††^	1854	0.16	0.25	2176
50–59	0.34 ^***, †††^	0.27 ^***, †††^	1586	0.16	0.27	1855
60–69	0.29 ^*, †^	0.20 ^***, †††, aaa^	999	0.20	0.20 ^*^	1011
70–74	0.29	0.30 ^**, ††^	269	0.19	0.14 ^*,a^	259

Group significantly different from the corresponding group aged 25–29 years: ^*^p < 0.05, ^**^p < 0.01, ^**^p < 0.001. Group significantly different from the corresponding group aged 30–39 years: ^†^*p* < 0.05, ^††^*p* < 0.01, ^†††^*p* < 0.001. Group significantly different from the corresponding group aged 40–49 years: ^a^*p* < 0.05, ^aa^*p* < 0.01, ^aaa^*p* < 0.001. In these comparisons, BMI (kg/m^2^), smoking (cigarettes/day) and ethanol intake (drinks/week) were used as covariates, as appropriate.

## Abbreviations

BMI: body mass index
GGT: gamma-glutamyltransferase
CI: confidence interval
GSH: glutathione
MONICA: Multinational Monitoring of Trends and Determinants in Cardiovascular Diseases
WHO: World Health Organization
